# Revista Brasileira de Medicina do Trabalho 2020-2022: challenges and advances

**DOI:** 10.47626/1679-4435-2022-203

**Published:** 2023-02-03

**Authors:** Andrea Franco Amoras Magalhães, Mírian Perpétua Palha Dias Parente

**Affiliations:** Revista Brasileira de Medicina do Trabalho’s Editors-in-Chief

As the management of the current Board of Directors of Associação Nacional de Medicina do Trabalho (ANAMT) comes to an end, as well as our time as Editors-in-Chief at Revista Brasileira de Medicina do Trabalho (RBMT), we will briefly summarize the changes that have been implemented in the Journal since 2020. Our goal is to further strengthen RBMT’s role as an important channel for disseminating scientific contributions that promote the improvement of working conditions and environments and the quality of life of health care professionals and workers in general.

Initially, we reformulated the Editorial Board to make it more comprehensive both nationally and internationally. To accelerate and improve editorial production, we decided to change suppliers and, as of volume 18, issue 2, 2020, we started working with Scientific Linguagem, a reference company in the publishing market that works with journals indexed in SciELO and other databases.

We have also been facing the consequences of the new coronavirus (SARS-CoV-2) pandemic and its variants since March 2020. These were difficult times, especially for health care professionals who worked to exhaustion in hospitals and other places caring for patients infected by COVID-19. But this tragedy also promoted learning and the discussion of new topics related to occupational medicine. RBMT encouraged, with a specific volume edition (20.1), the publication of articles related to COVID-19, with the objective of further disseminating the benefits of vaccination and measures aimed at reducing the risk of exposure to the virus.

In this new phase, the Journal has been significantly improved according to the best publishing practices. Some improvements are listed below.

- The website was redesign, with regular content updates.
- As of volume 18, issue 2, 2020: inclusion of ORCID iDs in all articles (as many as possible, but at least one author with ORCID per article).
- Review and standardization of data related to editors and members of the Editorial Board, including affiliation and country.
- Thorough proofreading and translation of article texts.
- Redesign of all figures.
- Working together with authors to provide more specific table titles.
- As of volume 18, issue 3, 2020: funding information and conflicts of interest were moved from the header to the title page footer to avoid space restrictions and header pollution.
- Inclusion of “How to cite” to help authors cite articles correctly.
- As of volume 18, issue 4, 2020: removal of copyright information (©) from the last page of the article, given that the journal does not collect copyright (articles are open access under the CC-BY license).
- As of volume 19, issue 2, 2021: reformulation of the Instructions for Authors page, including a clear indication of the journal’s mission.
- As of volume 19, issue 3, 2021: inclusion of authors’ contributions, as recommended by the Contributor Roles Taxonomy (CRediT).
- Increased number of reviewers and authors with a foreign affiliation.
- Journal registration on digital platforms such as Facebook, Instagram, and Twitter for increased reach of communication and dissemination.

Finally, in August 2022, we submitted an application for indexing RBMT in SciELO Brazil database. We are confident that the application will be approved, which will further insert RBMT in the national and international scientific community, valuing not only the journal, but all those who contribute with great dedication to occupational medicine in Brazil and Latin America.

